# Clavicle fractures and high‐grade acromioclavicular joint injuries do not affect posture and thoracic kyphosis in young adults: A prospective comparative study

**DOI:** 10.1002/jeo2.70821

**Published:** 2026-07-03

**Authors:** Jan‐Marek Meyer, Richard Julius Freytag, Ismaiel Al‐Ramadani, Mohammad Masoud, Jonathan Sommer, Jakob Zapatka, Alessandra Menon, Davide Cucchi

**Affiliations:** ^1^ Klinik für Orthopädie und Unfallchirurgie Universitätsklinikum Bonn Bonn Deutschland; ^2^ Laboratory of Applied Biomechanics, Department of Biomedical Sciences for Health Università degli Studi di Milano, Via Mangiagalli 31 Milan Italy; ^3^ U.O.C. 1° Clinica Ortopedica, ASST G. Pini‐CTO, Piazza Cardinal Ferrari 1 Milan Italy

**Keywords:** acromioclavicular joint dislocation, clavicle fracture, kyphosis, posture, rasterstereography, spinal alignment

## Abstract

**Purpose:**

Clavicle fractures and high‐grade acromioclavicular joint injuries are frequently associated with altered shoulder‐girdle function, but their impact on spinal posture remains unclear. This study aimed to evaluate whether these periclavicular injuries are associated with clinically relevant alterations in thoracic or cervical spinal alignment that would justify targeted spinal assessment or intervention.

**Methods:**

In this prospective comparative study, 45 patients with periclavicular injuries and 52 age‐matched healthy controls were assessed using rasterstereography to quantify spinal alignment in the sagittal (cervical plumb line distance, kyphosis and trunk inclination angles) and coronal planes (lateral deviation and plumbline deviation). The primary outcome was thoracic kyphosis angle. Subgroup analyses were performed based on injury chronicity (acute vs. subacute) and side dominance.

**Results:**

No significant differences in thoracic kyphosis or coronal/sagittal spinal parameters were observed between patients and controls, leading to rejection of the study hypothesis. Injury chronicity and side dominance did not influence outcomes.

**Conclusions:**

Periclavicular injuries are not associated with increased thoracic kyphosis on static posture assessment. These findings suggest that routine concern for structural thoracic kyphosis after clavicle fractures or high‐grade acromioclavicular joint injuries may be unwarranted in the context of static assessment.

**Level of Evidence:**

Level II, prospective comparative study.

**Abbreviations**
C7, VP: 7th cervical vertebra, Vertebra prominens
DM: midpoint of lumbar dimples
ICT: cervical‐thoracic inflection point
ITL: thoracic‐lumbar inflection point
L4: 4th lumbar vertebra
RMS: root mean square
T12: 12th thoracal vertebra

## INTRODUCTION

Arm movement relies on force transmission from the spine to the humerus through the shoulder girdle, requiring coordinated function across the entire kinetic chain, including the spine [18]. Disorders of the trunk or shoulder can disrupt arm motion, while upper‐arm and shoulder pathologies may also be expressed through compensatory changes in the trunk and spine [6, 25].

For example, pain arising from shoulder‐girdle injuries may reduce the contractility of the dorsoscapular muscle groups, resulting in altered scapular control and a posterior‐superior shift of the glenohumeral joint′s contact points [3]. These kinds of protective mechanisms are observed after clavicle injuries, where compensatory postures manifest as scapulothoracic dyskinesis [11, 15, 24].

In clinical practice, a frequently, yet insufficiently quantified observed consequence of shoulder‐girdle trauma is an ‘apparent’ increase in thoracic kyphosis, possibly associated with localised pain or tenderness in the paravertebral musculature. This presentation may arise either from altered scapular positioning (e.g., protraction), resulting in a ‘functional’ or false kyphosis, or from weakness of the thoracolumbar musculature, leading to a transient or permanent structural kyphosis of the thoracic spine [1, 26].

Patients with periclavicular injuries (injuries to the clavicle and acromioclavicular joint) may present with a visual impression of increased thoracic kyphosis. However, it remains unclear whether this finding reflects a measurable alteration in static spinal alignment. Clarifying this distinction is clinically relevant, as excessive thoracic kyphosis is associated with adverse long‐term outcomes and may influence rehabilitation priorities, particularly in active populations [12]. To date, no studies have systematically evaluated static spinal alignment following clavicle fractures or high‐grade acromioclavicular joint injuries [10, 11, 12]. Therefore, the aim of this prospective comparative study is to investigate whether periclavicular injuries induce clinically relevant alterations in static spinal alignment that would justify targeted spinal assessment or modification of rehabilitation strategies.

The hypothesis of this prospective comparative study is that patients with periclavicular injuries have an increased mean thoracic kyphosis angle (defined as the angle between the point of cervical‐thoracic inflection and that of the thoracic‐lumbar inflection) greater than 6°, when compared with age‐matched healthy controls.

## METHODS

### Design

All investigations were performed at the Department of Orthopedics and Trauma Surgery of the University of Bonn in the years 2021 to 2024. The study protocol was approved by the ethical committee of the medical faculty of the University of Bonn (Medizinischen Fakultät, Rheinischen Friedrich‐Wilhelms‐Universität Bonn, No. ID 419/19 + Amendment 20 May 2021). Written informed consent was obtained from all the participants. The STROBE Checklist was followed for result presentation.

### Criteria of inclusion and exclusion

Adult patients suffering from periclavicular injuries encompassing clavicle fractures and high‐grade acromioclavicular joint dislocations (Rockwood IIIb and V) were considered for inclusion. An upper age limit of 45 years was applied to minimise the influence of degenerative spinal changes. This threshold was selected based on previous reports demonstrating a high prevalence of age‐related alterations, such as vertebral body osteophytes, beginning in the late forties [17].

The comparison group consisted of healthy volunteers without any current or previous pathology of the shoulder girdle or upper limb, and without deformities of the trunk or spine. In line with the patient group, an upper age limit of 45 years was applied. Additionally, individuals engaged professionally in asymmetrical upper‐limb sports (e.g., fencing, tennis) were excluded to avoid bias related to unilateral muscular adaptation. Dyskinesis, which is commonly observed in asymptomatic individuals, particularly young, active people (prevalence ranging from 8.5% to 92%, and average of 48% in asymptomatic individuals) was not considered an exclusion criterion [19].

### Outcome measures

Rasterstereography (Formetric® DiCam, DIERS International GmbH) was used to assess spinal postural alignment in the coronal and sagittal planes. This is a well‐established, noninvasive method that does not involve radiation exposure and is used to generate a three‐dimensional image of the bony structures of the trunk. It is widely employed to monitor the progression of scoliosis and has demonstrated excellent precision in this field [9, 22].

**Figure 1:**
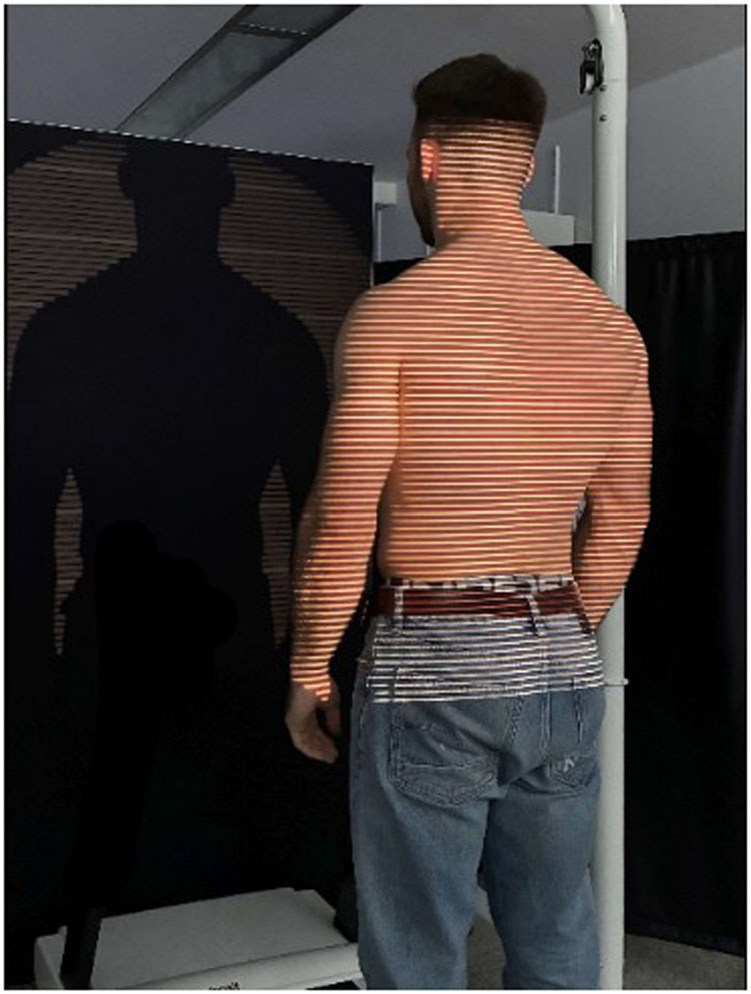
Clinical setup of the study: a grid pattern is projected onto the participant′s back surface.

Using a projector‐camera‐system a grid of parallel light rays is projected onto the back surface of the standing patient (Figure 1).

**Figure 2:**
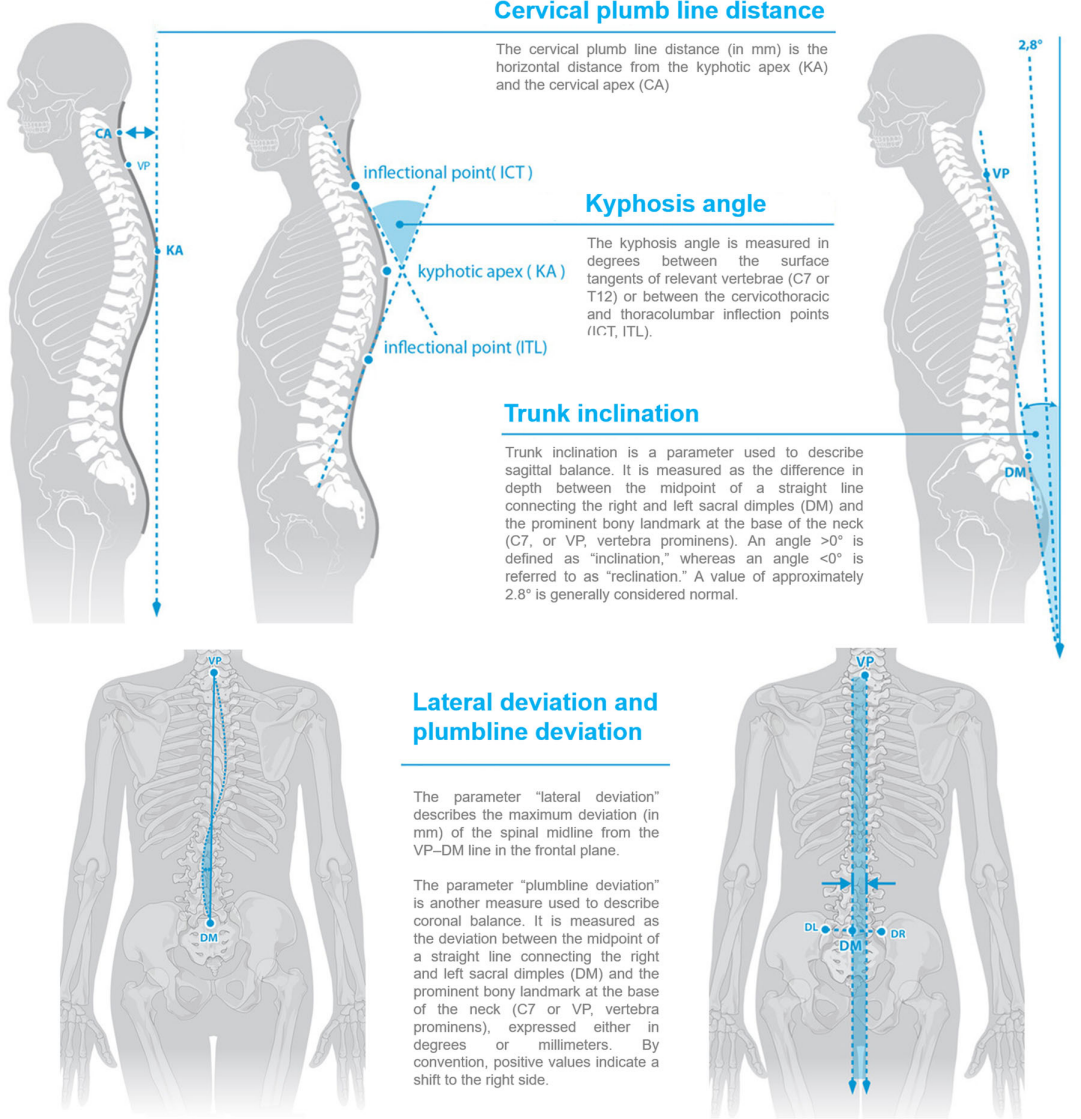
Graphic summary of the measured parameters in the sagittal and coronal projection. Courtesy of DIERS International GmbH, modified with permission; an extensive description of the study parameters is provided in the supplementary materials. C7, 7th cervical vertebra; DM, midpoint of lumbar dimples; ICT, cervical‐thoracic inflection point; ITL, thoracic‐lumbar inflection point; VP, Vertebra prominens; T12, 12th thoracal vertebra.

The distortion of the projected lines is recorded by a camera positioned above the projector. Using optical triangulation, a topographic surface map of the trunk is generated (rasterstereography). The associated software (Formetric 4Dmotion®Lab, DIERS International GmbH) reconstructs a three‐dimensional model of the underlying bony structures and calculates multiple spinal alignment parameters [5, 7]. Figure 2 offers a graphic summary of all study parameters, whereas a more detailed description of the measurement procedures, along with the complete list of parameters, is provided in the Supplementary Materials. Posture measurements were performed by multiple trained examiners following a standardised measurement protocol and without the use of reflective markers [8]. Previous studies already demonstrated high interobserver and intraobserver reliability, as well as consistent reproducibility across repeated measurements, independent of body mass index (BMI) and without the need for marker‐based correction [4, 8, 16, 21].

### Statistical analysis

Demographic data, as well as data regarding diagnosis and treatment of clavicle or acromioclavicular injuries and rasterstereography data, were entered into a spreadsheet for analysis. Statistical analysis was performed using GraphPad Prism v 6.0 software (GraphPad Software Inc.) and Microsoft Excel (Microsoft Corporation). Data distribution was assessed using the Shapiro–Wilk normality test before selecting the statistical tests. Continuous variables are presented as mean ± standard deviation (SD) for normally distributed data and as median with first and third quartiles [Q1–Q3] for nonnormally distributed data. Between‐group differences for continuous variables were analysed using the unpaired Student's *t*‐test or the Mann–Whitney *U*‐test, as appropriate according to the characteristics of the data distribution. Categorical variables are expressed in numbers of cases and frequencies; their differences were tested using the chi‐ squared test or Fisher′s exact test. For all analyses, the significance level was set at *p*‐value lower than 0.05. A post hoc Bonferroni correction was applied, taking into account the number of independent tests performed. An a priori sample size calculation was performed based on pilot data. The primary outcome parameter was the mean thoracic kyphosis angle. Based on the observed pilot values (patients: 55°, healthy controls: 49°) and an assumed SD of 10°, the study was powered to detect a between‐group difference of 6°. With a two‐sided alpha level of 0.05 and a power of 80%, the required total sample size was 88 participants (44 per group).

The following two‐sample comparisons were performed:

#### Primary analysis

Comparison of all healthy controls with all patients (upper age limit 45 years).

#### Secondary analysis

- Acute versus subacute injuries, using a 12‐week cutoff.
- Injuries of the dominant versus nondominant side, based on handedness relative to the injured side.

**Figure 3:**
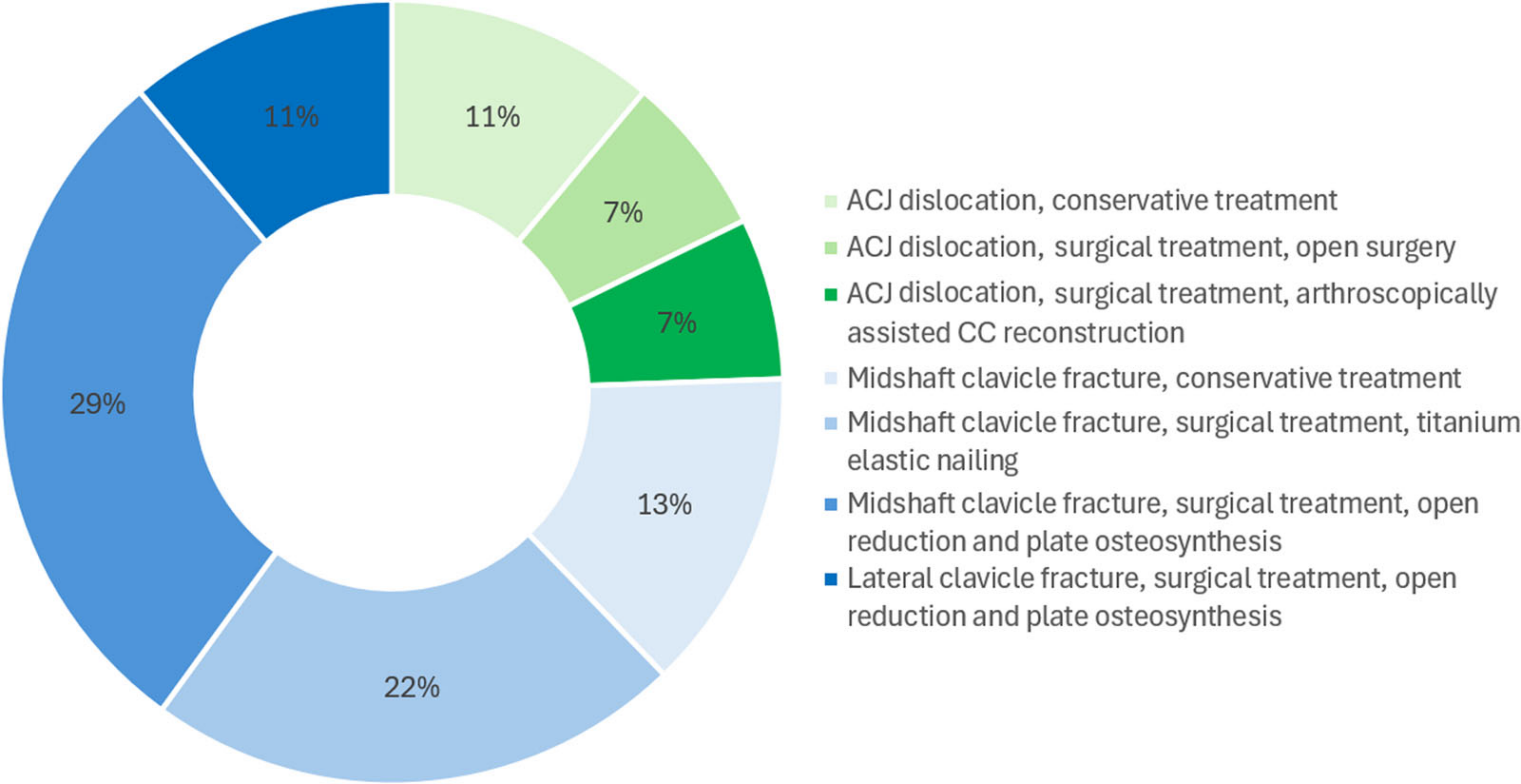
Pie chart illustrating the distribution of specific pathologies and treatments across the study population. ACJ, acromioclavicular joint; CC, coracoclavicular ligament.

Patient assessments were performed at multiple timepoints, considering the day of injury (for conservatively treated patients) or the day of surgical intervention as *t*_0_. The cutoff between subgroups was set at 12 weeks, corresponding to the transition from the proliferative to the remodelling phase of bone and tendon healing [13]. No further subgroup stratification based on injury patterns was performed, given their comparable impact on the trunk and spine, as well as the considerable heterogeneity of the included pathologies and treatments (Figure 3).

## RESULTS

A total of 52 healthy controls (mean age: 26.3 ± 3.3 years, females: 42%; right‐hand dominance: 92%) and 45 patients (mean age: 28.7 ± 8.6 years, females: 29%; right‐hand dominance: 96%), were enrolled. Specific pathology‐related data are provided in Figure 3.

No significant differences were observed between the two groups with respect to gender distribution, BMI or handedness. Likewise, no significant differences were found in the primary outcome or in any of the additional parameters characterising spinal postural alignment in the coronal or sagittal planes (Table 1). Consequently, the study hypothesis could be rejected.

**Table 1:** Comparison of demographic data and of rasterstereographic spinal parameters between healthy controls and patients (<45 years of age) suffering from periclavicular injuries encompassing clavicle fractures and high‐grade acromioclavicular joint dislocations (Rockwood IIIb and V).

Parameter	Healthy controls	Patients	*p*‐Value
Age (years)	26.3 ± 3.3	28.7 ± 8.6	n.s.
Gender (F/M ratio)	0.42/0.58	0.29/0.71	n.s.
Dominant side (L/R ratio)	0.08/0.92	0.04/0.96	n.s.
Trunk inclination C7‐DM (°)	1.5 [0.7–2.2]	1.9 [0.8−3.6]	n.s.
Trunk inclination C7‐DM (mm)	11.2 [5.4–20.4]	15.8 [7.2–30.7]	n.s.
Plumbline deviation C7‐DM (°)	0.7 [0.4–1.3]	1.1 [0.4–1.6]	n.s.
Plumbline deviation C7‐DM (mm)	6.4 [3.7–10]	6.0 [2.8–12.6]	n.s.
Lateral deviation RMS	4.0 [2.7–5.7]	3.7 [2.4–5.2]	n.s.
Lateral deviation right (mm)	6.2 [4.2–9.0]	5.9 [2.5–9.4]	n.s.
Lateral deviation left (mm)	1.9 [0.4–4.2]	1.5 [0.0–4.4]	n.s.
Kyphosis angle C7‐T12 (°)	43.7 ± 8.3	46.1 ± 7.1	n.s.
Cervical plumb line distance (mm)	60.2 ± 16.8	66.4 ± 18.3	n.s.
Kyphosis angle ICT‐ITL (°)	49.5 ± 7.9	52.6 ± 8.8	n.s.
Kyphosis angle C7‐ITL (°)	46.0 ± 8.1	49.1 ± 7.9	n.s.

*Note*: Normally distributed variables are presented as mean ± standard deviation (SD), and nonnormally distributed variables as median [Q1–Q3].

Abbreviations: C7, C7 Vertebra; DM, Midpoint of lumbar dimples; ICT, cervical‐thoracic inflection; ITL, thoracic‐lumbar inflection; n.s., nonsignificant; RMS, root mean square; T12, 12th thoracal vertebra.

### Subgroup analyses

Comparing rasterstereography results of patients obtained in an acute and subacute phase (time from injury or operation: 46.0 ± 16.8 vs. 182.0 ± 119.9 days, *p* < 0.0001) confirmed similar demographic data and outcome measures between the groups, except for a tendency towards a small difference in left lateral deviation, which was lower in acutely injured patients (median −2.2 mm) (Table 2).

**Table 2:** Comparison of rasterstereographic spinal parameters between patients with acute and subacute periclavicular injuries.

Parameter	Acute	Subacute	*p*‐Value
Time from injury or operation (days)	46.0 ± 16.8	182.0 ± 119.9	<0.0001
Trunk inclination C7‐DM (°)	1.8 [0.6–3.0]	1.6 [0.8–2.5]	n.s.
Trunk inclination C7‐DM (mm)	15.1 [5.6–25.0]	12.6 [6.4–21.4]	n.s.
Plumbline deviation C7‐DM (°)	0.7 [0.4–1.4]	1.1 [0.5–1.6]	n.s.
Plumbline deviation C7‐DM (mm)	4.3 [2.9–12.2]	8.7 [4.3–13.4]	n.s.
Lateral deviation RMS	4.6 [2.8–6.4]	2.9 [1.9–4.7]	n.s.
Lateral deviation right (mm)	7.3 [2.5–12.3]	4.9 [3.1–7.1]	n.s.
Lateral deviation left (mm)	0.2 [0.0–3.1]	2.4 [0.6–4.8]	0.042 (n.s.)
Kyphosis angle C7‐T12 (°)	45.3 ± 6.3	45.0 ± 8.0	n.s.
Cervical plumb line distance (mm)	66.7 ± 16.7	67.2 ± 18.9	n.s.
Kyphosis angle ICT‐ITL (°)	51.8 ± 8.0	50.7 ± 9.5	n.s.
Kyphosis angle C7‐ITL (°)	48.5 ± 7.2	47.7 ± 8.8	n.s.

*Note*: Normally distributed variables are presented as mean ± standard deviation (SD), and nonnormally distributed variables as median [Q1–Q3]. *p*‐values after Bonferroni correction are showed in brackets.

Abbreviations: C7, C7 Vertebra; DM, Midpoint of lumbar dimples; ICT, cervical‐thoracic inflection; ITL, thoracic‐lumbar inflection; n.s., nonsignificant; RMS, root mean square; T12, 12th thoracal vertebra.

This difference persisted when comparing patients with acute lesions to the healthy control, although its clinical relevance remains uncertain. In the comparison between the subacute subgroup and the healthy control group, no parameters showed significant differences.

The involvement of the dominant or nondominant side had no effect on any of the study parameters.

## DISCUSSION

The main finding of this study is that patients with periclavicular injuries did not show a significant difference in mean thoracic kyphosis angle compared with healthy controls. This supports the interpretation that the postural changes commonly observed in these patients are functional, shoulder‐driven adaptations rather than structural alterations of the thoracic spine.

From a clinical perspective, these findings support prioritising rehabilitation strategies that target shoulder‐girdle mechanics, scapular control and muscular balance rather than thoracic spinal alignment in the absence of specific spinal symptoms; however, they should be interpreted with caution, as only static alignment was assessed and dynamic compensatory mechanisms were not evaluated. These findings highlight that functional and dynamic alterations of the shoulder girdle, such as pain‐related changes in scapular control, posterior‐superior shifts of the glenohumeral joint contact points or scapulothoracic dyskinesis, may play a greater role in generating the apparent ‘functional’ kyphosis observed in these patients rather than changes in the postural control of the spine, findings which appear consistent with previous research focused on scapulothoracic adaptive changes after shoulder girdle injuries [3, 11, 24]. Furthermore, a recent study investigating postoperative spinal static and dynamic changes after rotator cuff repair surgery suggested an association with kyphotic posture, supporting the idea that shoulder‐girdle injuries can be linked to spinal posture changes [25]. Clinically, these findings may support attention to muscular function and scapular control rather than routine assumptions of altered spinal alignment after periclavicular injuries.

The hypothesis underlying this study was that periclavicular injuries might be associated with measurable postural changes of the spine, reflecting a link between functional impairment of the shoulder girdle and compensatory adaptations of the thoracic spine [6, 18]. This premise was based on the frequent clinical observation that patients with periclavicular injuries often present an apparent thoracic hyperkyphosis and that this condition is frequently observed yet insufficiently quantified also in patients with shoulder pain of other origins [1]. Given that thoracic hyperkyphosis is known to have long‐term detrimental functional and health‐related consequences, early detection of such posttraumatic adaptations was considered particularly important for the development of targeted rehabilitation strategies, especially in athletic populations [12]. A critical distinction must be made between an apparent or functional kyphosis, where sagittal spinal alignment remains unchanged but alterations in the paravertebral and periscapular musculature and scapular positioning create a visually exaggerated kyphotic posture, and a structural kyphosis, characterised by a transient or permanent measurable alteration of spinal alignment, since these two entities are approached in different ways by physical therapists. At present, only a few rehabilitation protocols for periclavicular injuries are available [2], and these generally do not include specific measures to address spinal alterations. In contrast, evidence‐based exercise programs targeting upper crossed syndrome (also referred to as ‘upper cross’ syndrome) are well established [23].

Therapeutic concepts derived from upper crossed syndrome programs may be of interest in this specific context and in the younger population, in particular exercises addressing cervical and scapular postural control, although treatment effects were not assessed in the present study. This interpretation is in line with current evidence showing that specific training programs can effectively reduce both thoracic kyphosis and associated postural patterns [23].

The strengths of this study are its prospective comparative design with clearly defined inclusion and exclusion criteria, including an upper age limit to minimise the confounding influence of degenerative spinal changes and the use of a well‐established, reproducible and radiation‐free method for assessing spinal alignment [10, 14, 20]. Nevertheless, some limitations must be acknowledged. First, the study cohort was relatively small, particularly within subgroups and included patients with heterogeneous presentations of periclavicular pathology, which may have limited the ability to detect subtle or clinically relevant postural differences; some of the minimal alterations detected in subgroup analyses, which could reflect some degree of compensatory postural adaptation, will require larger cohorts to be confirmed. Second, while rasterstereography provides excellent noninvasive surface‐based assessment, it remains an indirect measure of spinal alignment: it may not fully capture underlying structural changes as radiographic methods do and currently lacks the ability to quantify scapular positional and kinematic parameters, such as protraction/retraction, rotation and tilt; furthermore, the considerable upfront costs and requirement for specialised software updates remain significant barriers, restricting the widespread diffusion of this diagnostic approach, particularly in low‐resource settings. Third, the cross‐sectional nature of the assessment precludes evaluation of temporal changes or causality between periclavicular injuries and spinal posture. Fourth, although participants with professional asymmetrical sport exposure were excluded, variations in recreational activity levels were not fully controlled, which may introduce variability in postural adaptations. Finally, the study focused on static posture; dynamic postural alterations, which may be more relevant to shoulder girdle compensation, were not captured, limiting the generalisability of findings to everyday or sport‐specific movements.

## CONCLUSION

Periclavicular injuries are not associated with increased thoracic kyphosis on static posture assessment. These findings suggest that routine concern for structural thoracic kyphosis after clavicle fractures or high‐grade acromioclavicular joint injuries may be unwarranted in the context of static assessment.

## AUTHOR CONTRIBUTIONS

**Jan‐Marek Meyer**: conceptualization, data collection, manuscript draft, statistical analysis, figures and tables. **Richard Julius Freytag**: data collection, manuscript revision, supervision. **Ismaiel Al‐Ramadani**: data collection. **Mohammad Masoud**: data collection. **Jonathan Sommer**: data collection. **Jakob Zapatka**: data collection. **Alessandra Menon**: statistical analysis, tables. **Davide Cucchi**: conceptualization, manuscript revision, supervision.

## FUNDING INFORMATION

The authors have no funding to report.

## CONFLICT OF INTEREST STATEMENT

Davide Cucchi is an associate editor of JEO; the remaining authors declare no conflicts of interest.

## ETHICS STATEMENT

IRB approval: Ethikkommission an der Medizinischen Fakultät der Rheinischen Friedrich‐Wilhelms‐Universität Bonn, Nr. ID 419/19 + Amendment 20 May 2021. Written informed consent was obtained from all human participants.

## Supporting information

Supporting File 1
