# Supplementary material for: Clavicle fractures and high‐grade acromioclavicular joint injuries do not affect posture and thoracic kyphosis in young adults: A prospective comparative study
Source: J Exp Orthop. 2026 Jul 3;13(3):e70821. doi: 10.1002/jeo2.70821 (PMC13331606; doi:10.1002/jeo2.70821)
Supplement: Supplementary file 1 — Supporting File 1 [file JEO2-13-e70821-s001.docx]

**Supplementary Materials:**

1. Rasterstereography:

The rasterstereography methodology is based on the principle of light-optical triangulation. Using a projector, a horizontal grid of parallel lines is projected onto the back surface of standing participants. The projected grid is recorded by a camera positioned above the projector. The distortion of the projected lines provides information on the three-dimensional surface structure of the back. Measurements were performed using the Formetric® DiCam projector-camera system (DIERS International GmbH, Christof-Ruthof-Weg 6, 55252 Wiesbaden, Germany), with a resolution of 7,500 points and a reconstruction error of < 0.2 mm (Drerup & Hierholzer, 1994). The length of the triangulation baseline (the distance between the projector and the cameras) and the angular positions of the projector and cameras relative to this baseline are known, allowing precise calculation of the three-dimensional coordinates of all surface points. Using the proprietary software formetric 4Dmotion®Lab, characteristic anatomical landmarks, such as the C7 vertebra, and symmetry lines, including the spinal midline, are identified based on curvature distribution. For data reproducibility and interpretation, all surface points are referenced to the C7 vertebra (Horn, 2010). In dynamic rasterstereographic motion analyses, reference points such as the C7 vertebra and the left and right lumbar dimples are marked on the back surface using reflective markers. In static analyses, such marking is not required, as the software can calculate these anatomical landmarks with sufficient accuracy (Drerup & Hierholzer, 1987). Based on these reference points, a topographic map of the back surface can be generated independently of the participant’s position relative to the camera, allowing the calculation of multiple interpersonally comparable postural parameters.


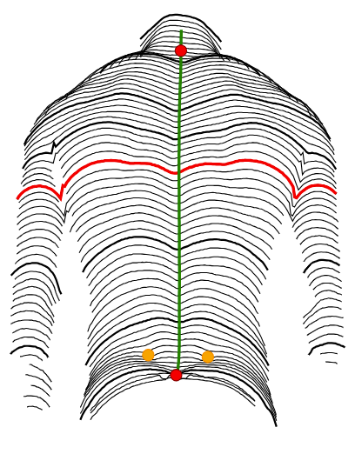

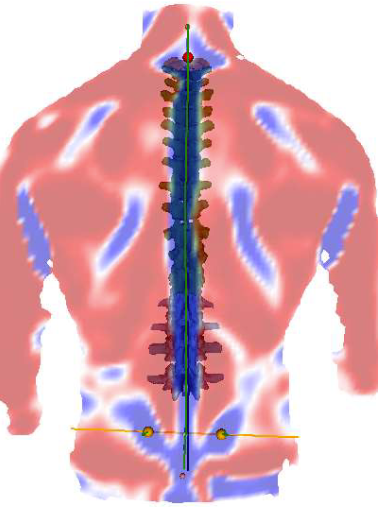

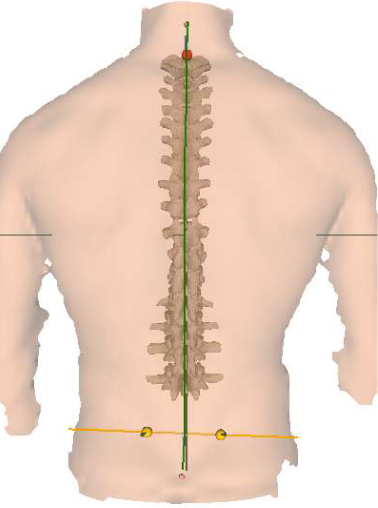


*Figure 1: Examples of the generated 3D-Models of the trunk. Courtesy of DIERS International GmbH.*

2. Parameters:

In the sagittal projection, parameters including trunk inclination, kyphosis angles, and cervical plumb line distance were assessed. Trunk inclination describes the ventral or dorsal displacement of the midpoint between the two lumbar dimples relative to the plumb line originating from the C7 vertebra.

Thoracic kyphosis was quantified using three different angular measurements. The ICT–ITL angle was defined as the angle between the tangent line at the cervicothoracic inflection point and the tangent line at the thoracolumbar inflection point. The VP–ITL angle was defined as the angle between the tangent line through the C7 vertebra and the tangent line at the thoracolumbar inflection point. The VP–T12 angle was defined as the angle between the tangent line through the C7 vertebra and the tangent line at the level of T12.

The cervical plumb line distance describes the horizontal distance between the most anterior cervical apex and the plumb line passing through the most posterior apex of thoracic kyphosis. In addition, the “cervical plumb line distance (C7)” was defined as the distance between the C7 vertebra and the same plumb line.

In the frontal projection, parameters including lateral offset and side deviation were assessed. Lateral offset describes the horizontal displacement of the midpoint between the two lumbar dimples from the plumb line through C7. Side deviation represents the maximum lateral deviation of any vertebral apex between C7 and L4. Three values were derived: the maximum distance from the C7 plumb line to the most laterally deviated vertebral apex on the left, the corresponding distance on the right, and the total amplitude between the leftmost and rightmost deviations (Horn, 2010).

Validation:

Meta-analyses have demonstrated that static rasterstereography represents a reliable and valid alternative to conventional radiographic imaging for the assessment of spinal alignment. For thoracic kyphosis angle measurements, an average correlation coefficient of r = 0.92 for reliability and r = 0.75 for validity has been reported across 12 studies (Krott et al., 2020).

The reliability of our measurements is consistent with previously published reference values. In healthy controls, the mean thoracic kyphosis angle (ICT–ITL) measured in this study was 49.5° ± 7.9°, which closely corresponds to values reported by Schröder et al. (48° ± 9°) and Kaps et al. (50.03° ± 8.18°) (Schröder et al., 2011; Kaps et al., 2023).

References:

Drerup, B., & Hierholzer, E. (1987). Movement of the human pelvis and displacement of related anatomical landmarks on the body surface. *Journal of Biomechanics*, *20*(10), 971–977. https://doi.org/10.1016/0021-9290(87)90326-5

Drerup, B., & Hierholzer, E. (1994). Back shape measurement using video rasterstereography and three-dimensional reconstruction of spinal shape. *Clinical Biomechanics*, *9*(1), 28–36. https://doi.org/10.1016/0268-0033(94)90055-8

Horn, T. (2010). *Optische Wirbelsäulen-vermessung Informationen zur Auswertung*.

Kaps, D., Siebers, H. L., Betz, U., Pfirrmann, D., Eschweiler, J., Hildebrand, F., Betsch, M., Huthwelker, J., Wolf, C., Drees, P., & Konradi, J. (2023). Creation and Evaluation of a Severity Classification of Hyperkyphosis and Hypolordosis for Exercise Therapy. *Life*, *13*(6). https://doi.org/10.3390/life13061392

Krott, N. L., Wild, M., & Betsch, M. (2020). Meta-analysis of the validity and reliability of rasterstereographic measurements of spinal posture. *European Spine Journal*, *29*(9), 2392–2401. https://doi.org/10.1007/s00586-020-06402-x

Schröder, J., Stiller, T., & Mattes, K. (2011). Referenzdaten in der Wirbelsäulenformanalyse: Annäherung an eine Majoritätsnorm und Abweichungen bei unspezifischen Rückenschmerzsyndromen. *Manuelle Medizin*, *49*(3), 161–166. https://doi.org/10.1007/s00337-011-0831-1
